# FMRP restoration in parvalbumin interneurons: A circuit-specific improvement of visual learning in fragile X syndrome

**DOI:** 10.1016/j.isci.2025.114132

**Published:** 2025-11-21

**Authors:** Sanghamitra Nareddula, Violeta Saldarriaga, Xinwan Hu, Paige Alyssa Edens, Mia Fehlinger, Alexander A. Chubykin

**Affiliations:** 1 Department of Biological Sciences, Purdue Institute for Integrative Neuroscience, Purdue Autism Research Center, Purdue University, West Lafayette, IN 47907, USA

**Keywords:** genetics, neuroscience

## Abstract

Autism spectrum disorders (ASDs) are neurodevelopmental disorders characterized by impaired sensory processing and learning disabilities, of which Fragile X syndrome (FX) is a leading genetic cause. Visual familiarity evokes persistent theta oscillations (4–8 Hz) in the primary visual cortex (V1) of mice, which are weaker and slower in *Fmr1* KO mice correlating with the decreased excitatory synaptic drive onto fast-spiking interneurons. To test if loss of *Fmr1* protein (FMRP) expression in parvalbumin-positive (PV+) fast-spiking interneurons is causal to impaired oscillations and learning, we restored FMRP selectively in PV+ interneurons (*Fmr1*cON/PV-Cre). Silicon probe recordings of V1 showed that targeted restoration rescued frequency and duration of oscillatory responses and improved direction tuning deficits seen in FX. These mice also exhibited improved performance in a visual learning task and decreased hyperactivity. Our findings support an important role of PV+ interneurons in visual learning and memory, suggesting promising avenues for circuit-specific therapeutic interventions in FX syndrome.

## Highlights

• Restoration of FMRP in PV+ interneurons improves visual memory in FX mice
• Direction tuning impairments are rescued in the CON mice
• CON mice perform better at visual discrimination tasks
• CON mice have reduced hyperactivity and improved social interaction behaviors

## Graphical abstract

**Figure:**
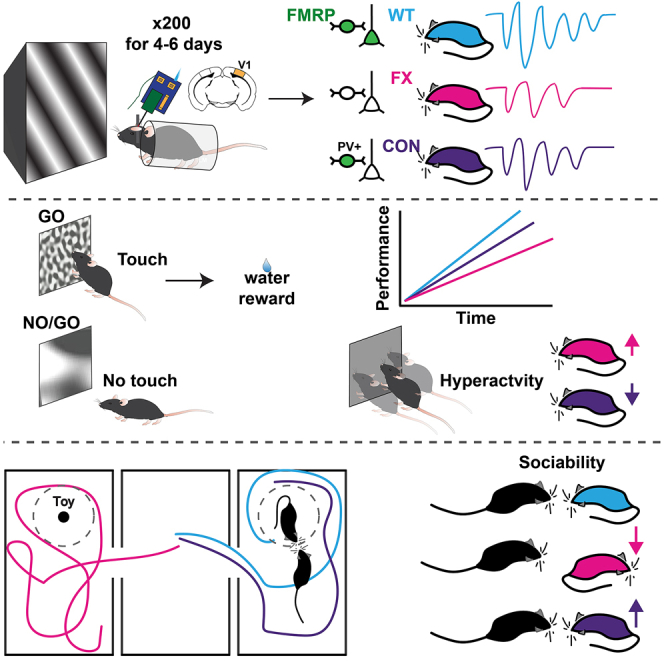

## Introduction

Fragile X syndrome (FX) is one of the leading genetic causes of autism spectrum disorders (ASDs) and intellectual disability (ID), resulting in impaired cognitive function and learning.^1^ It is caused by a single gene mutation in the Fragile X Messenger Ribonucleoprotein 1 (*Fmr1*) gene, resulting in hypermethylation and the corresponding loss of expression of the *Fmr1* protein (FMRP).^2^ Patients with FX have impairments in sensory perception, typically showing hypersensitivity to external stimuli.^1^^,^^3^ In *Fmr1* knockout (*Fmr1* KO) mice models of FX, studies have shown similar deficits in sensory processing, hypersensitivity, and perceptual learning.^4^^,^^5^^,^^6^ Deficits in the FX neural response have been associated with altered excitation-inhibition (E/I) balance of neural networks, documented in FX and ASDs.^7^^,^^8^^,^^9^ Functional, physiological, and structural impairments in interneurons have been widely noted in FX, likely contributing to the E/I imbalance.^4^^,^^5^^,^^6^^,^^8^^,^^9^^,^^10^^,^^11^ Parvalbumin-positive (PV+) neurons form the majority of the cortical inhibitory interneuron population, tightly regulating and synchronizing spike timing in excitatory cells, and are widely reported to be hypoactive and fewer in number in FX.^4^^,^^8^^,^^11^^,^^12^ FMRP is known to play a major role in neuronal synaptic translational regulation and is capable of directly interacting with ion channels, the loss of which results in the overexpression of synaptic proteins and affects the excitability of neurons and synaptic plasticity in neural circuits.^13^^,^^14^^,^^15^ Furthermore, the loss of FMRP in PV+ interneurons alone has been shown to cause behavioral deficits associated with FX.^16^ *Fmr1* KO mice have been shown to have delayed learning in a visual discrimination task corresponding to reduced function of PV+ interneurons and broader tuning in pyramidal cells.^4^ Visual experience evokes low-frequency persistent 4–8 Hz theta oscillations in V1 that are specific to the familiar stimulus.^17^^,^^18^^,^^19^ These evoked oscillations are lower in frequency, smaller in amplitude, and shorter in duration in FX mice.^20^^,^^21^ Directed information analysis of the visually evoked activity and synaptic circuit mapping in V1 identified several intracortical connectivity changes post-visual experience, which are attenuated in FX mice. Notably, strengthening of layer 5 pyramidal neuron projections onto layer 4 fast-spiking (L4 FS) interneurons post-visual experience, seen in V1 of WT mice, was lost in FX mice.^10^^,^^20^

In order to further understand the contribution of PV+ interneurons to visual memory encoding in V1, we used a unique transgenic line, *Fmr1*cON/PV-Cre (CON) mice,^22^ to conditionally express FMRP in PV+ interneurons and study the network response using electrophysiology and analyze their performance in a visual discrimination task. CON mice showed a partial rescue of visual experience-evoked neural responses in V1. Silicon probe recordings in V1 showed a partial increase in amplitude, a complete shift in frequency, longer durations, and increased oscillation cycles in CON mice compared to FX mice in local field potentials (LFPs) and unit population activity. Furthermore, direction tuning analysis showed an increase in selectivity index in WT and CON mice post-visual experience, which was absent in FX mice, indicating a rescue in tuning properties. Most notably, we discovered that CON mice performed significantly better at a Go/No-Go visual discrimination task compared to FX mice, reaching expert-level training score (TS) in a fewer number of days. Hyperactivity and social behavior impairments were also significantly alleviated in CON mice. Overall, the results from this study show that restoring FMRP in PV+ interneurons alone partially rescues visual experience-evoked theta oscillations in mice V1. This restoration of the network response translates to significant improvements in learning deficits to perceptual behavior tasks; shedding light on the role of PV+ interneurons in visual memory encoding, hyperactivity, and social interactions; and providing a potential avenue for therapeutic intervention for FX and phenotypically related ASD symptoms.

## Results

### LFP activity shows stronger visually evoked responses in CON mice

**Figure 1:**
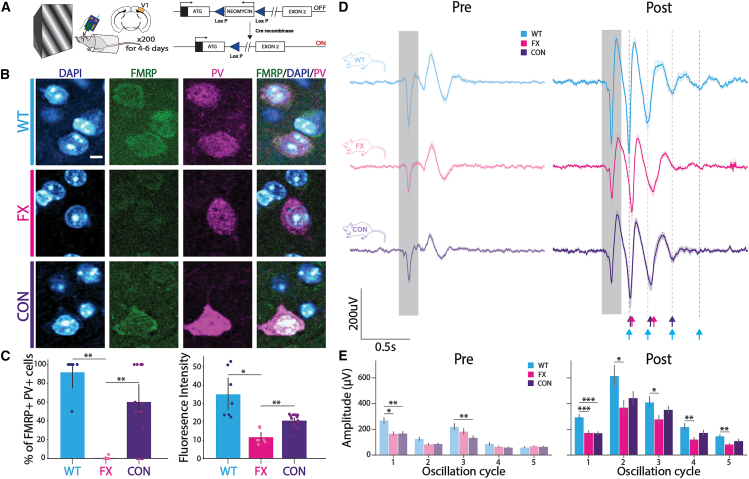
LFPs in CON mice have higher VEP amplitudes compared to FX mice (A) Left: schematic representation of passive visual experience paradigm and extracellular *in vivo* recordings in mice V1. Right: schematic representation of the *Fmr1* transgene in Fmr1cON mice lines crossed with Cre recombinase. (B) IHC showing expression of FMRP in WT, FX, and CON mice. Staining shows cell nuclei (blue), FMRP (green), and PV interneurons (magenta). Scale bars: 5 μm. (C) Left: IHC quantification showing percentage of PV+ cells containing FMRP. Right: mean fluorescence of FMRP in all PV+ cells (WT: *n* = 6 images from 3 mice, FX: *n* = 7 images from 3 mice, CON: *n* = 16 images from 3 mice). Mann-Whitney U test (left: WT vs. FX: *p* = 0.004, CON vs. FX: *p* = 0.009; right: WT vs. FX: *p* = 0.04, CON vs. FX: *p* = 0.005). (D) Trial averaged VEPs in V1 of WT (cyan), FX (magenta), and CON (indigo) mice. Trial averaged VEPs pre- (left; WT: *n* = 13 mice, FX: *n* = 15 mice, CON: *n* = 16 mice) and post- (right; WT: *n* = 12 mice, FX: *n* = 16 mice, CON: *n* = 13 mice) visual experience. Arrows indicate peaks of VEPs for each oscillation cycle for each strain. (E) VEP amplitudes across 5 oscillation cycles in WT (cyan), FX (magenta), and CON (indigo) mice pre- (left; WT: *n* = 13 mice, FX: *n* = 15 mice, CON: *n* = 16 mice) and post- (right; WT: *n* = 12 mice, FX: *n* = 16 mice, CON: *n* = 13 mice) visual experience. Two-way ANOVA (Factor1: strain; Factor2: oscillation cycle) significant interaction between strain and oscillation cycle on VEP amplitude for both pre- and post-visual experience (pre: F = 3.3, *p* = 0.0014; post: F = 2.13, *p* = 0.0035). Tukey HSD multiple comparisons of means across strains for both pre- and post-visual experience (pre: cycle 1: WT vs. FX: *p* = 0.034; WT vs. CON: *p* = 0.003; cycle 3: WT vs. CON: *p* = 0.035; post: cycle 1: WT vs. FX: *p* = 0.0; WT vs. CON: *p* = 0.0001; cycle 2: WT vs. FX: *p* = 0.024; cycle 3: WT vs. FX: *p* = 0.019; cycle 4: WT vs. FX: *p* = 0.005; cycle 5: WT vs. FX: *p* = 0.001). Data are presented as mean ± SEM. ∗*p* < 0.05, ∗∗*p* < 0.01, ∗∗∗*p* < 0.001.

**Figure 2:**
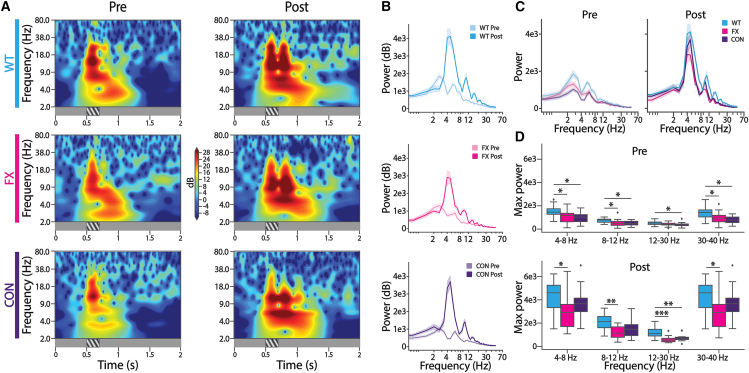
CON mice show an increase in power in the 4–8 Hz theta band range post-visual experience (A) Heat maps of time-frequency spectrograms of trial averaged V1 VEPs in WT (cyan), FX (magenta), and CON (indigo) mice pre- (left; WT: *n* = 13 mice, FX: *n* = 15 mice, CON: *n* = 16 mice) and post- (right; WT: *n* = 12 mice, FX: *n* = 16 mice, CON: *n* = 13 mice) visual experience. (B) Power spectrum analysis of trial averaged V1 VEPs across various frequency bands averaged within a time window of 0.7–2.0 s after stimulus onset pre- and post-visual experience within strains in WT (cyan, top), FX (magenta, middle), and CON (indigo, bottom) mice (pre; WT: *n* = 13 mice, FX: *n* = 15 mice, CON: *n* = 16 mice; post; WT: *n* = 12 mice, FX: *n* = 16 mice, CON: *n* = 13 mice). (C) Power spectrum analysis of trial averaged V1 VEPs across various frequency bands averaged within a time window of 0.7–2.0 s after stimulus onset shown across strains in WT (cyan, top), FX (magenta, middle), and CON (indigo, bottom) mice pre- (left; WT: *n* = 13 mice, FX: *n* = 15 mice, CON: *n* = 16 mice) and post- (right; WT: *n* = 12 mice, FX: *n* = 16 mice, CON: *n* = 13 mice) visual experience (D) Quantification of power spectrum analysis across various frequency bands of trial averaged V1 VEPs in WT (cyan), FX (magenta), and CON (indigo) mice. Maximum power within each frequency band calculated from power spectrum analysis shown in (C) averaged across mice pre- (top; WT: *n* = 13 mice, FX: *n* = 15 mice, CON: *n* = 16 mice) and post- (bottom; WT: *n* = 12 mice, FX: *n* = 16 mice, CON: *n* = 13 mice) visual experience. Mann-Whitney U test. (Pre: 4–8 Hz; WT vs. FX: *p* = 0.02, WT vs. CON: *p* = 0.008, 8–12 Hz; WT vs. FX: *p* = 0.017, WT vs. CON: *p* = 0.003, 12–30 Hz; WT vs. CON: *p* = 0.037, 30–40 Hz; WT vs. FX: *p* = 0.011, WT vs. CON: *p* = 0.0015, post: 4–8 Hz; WT vs. FX: *p* = 0.019, 8–12 Hz; WT vs. FX: *p* = 0.003, 12–30 Hz; WT vs. FX: *p* = 0.0004, WT vs. CON: *p* = 0.006, 30–40 Hz; WT vs. FX: *p* = 0.019.) ∗*p* < 0.05, ∗∗*p* < 0.01, ∗∗∗*p* < 0.001. Data are shown as boxplots with 25th, median, and 75th percentiles, and whiskers indicate min and max values.

To restore FMRP in PV+ interneurons only, we used a unique conditional restoration strain (*Fmr1*^loxP−Neo/y^) crossed with a PV-specific Cre line (PV-Cre^+/+^) and confirmed the restoration process using immunohistochemistry (IHC) (Figures 1A–1C).^22^ The *Fmr1*^loxP−Neo/y^ mouse line has a floxed neomycin cassette at intron 1 of the *Fmr1* gene. In the presence of Cre recombinase, the neomycin cassette is excised, and FMRP expression is restored to normal levels. Crossing *Fmr1*^loxP−Neo/y^ with PV-Cre^+/+^ conditionally restores FMRP in PV+ interneurons only, where Cre recombinase is expressed under the parvalbumin promoter (Figures 1A–1C). IHC quantification analysis showed a significant increase in FMRP levels and the number of PV+ interneurons containing FMRP in the conditional restoration strain, compared to *Fmr1* KO mice (Figure 1C). The study was performed blindly to the three genotypes: WT, *Fmr1* KO (FX), and *Fmr1*cON/PV-Cre (CON). Mice were placed in front of a monitor and head-fixed to ensure carefully controlled and reproducible visual stimulus perception. Mice were habituated to this setup for 3–4 days during which they viewed a gray screen. On the day of the experiment, 64-channel silicon probes were inserted into the primary visual cortex (V1), and recordings were made from one of the hemispheres, while the mice viewed 20 presentations of a 200-ms sinusoidal drifting grating stimulus (60°, 0.04 cycles/degree) interspersed with a gray screen. After these initial pre-training recordings, mice were familiarized with the stimulus after 200 presentations of the same drifting grating stimulus each day for 4–5 days. We then recorded from V1 of the other hemisphere, while the mice viewed 20 presentations of the drifting grating stimulus. LFPs were analyzed by selecting the channel with the most negative amplitude and averaging the response across all trials for each mouse. Prior to visual familiarity, averaged LFPs showed a single peak response during the visual stimulus time window (0.5–0.7 s) across all three strains (Figure 1D). Following visual experience, LFP activity showed stimulus-locked low-frequency theta oscillations, persisting long after the visual stimulus (Figure 1D), consistent with our previous research.^17^^,^^19^ Quantification of visually evoked potentials (VEPs) within each oscillation peak time window showed a significant decrease in amplitude across all five oscillation cycles between WT and FX mice (Figures 1D and 1E). This decrease in amplitude indicates an attenuated response in FX. We found no difference in VEP amplitude for oscillation cycles 2, 3, 4, and 5 between CON and WT mice (Figures 1D and 1E). However, the VEP amplitudes are not significantly higher in CON mice compared to FX (Figures 1D and 1E), possibly indicating an intermediate phenotype and a partial restoration of the strength of the oscillatory response. To investigate the power across various frequency bands (4–8, 8–12, 12–30, and 30–40 Hz) of the LFP activity, we performed time-frequency analysis and quantified the power during a post-visual stimulus time window (0.5–2.0 s) (Figures 2A–2D). Power spectra show that the theta band (4–8 Hz) power is significantly lower in FX mice compared to WT mice, while there is no significant difference between the CON and WT mice (Figure 2D). Though CON mice have higher theta band power compared to FX, this does not reach significance, further indicative of a partial increase in the familiarity evoked oscillatory response. Studies show that alpha (8–12 Hz) and gamma (30–40 Hz) frequencies are associated with visual information processing and sensory integration in mice.^23^^,^^24^ Here, we see that FX mice have decreased power, compared to WT mice, in both the alpha band (8–12 Hz) and the gamma band (30–40 Hz) (Figure 2D). CON mice show no significant differences in power compared to WT or FX mice in 4–8 Hz, 8–12 Hz and 30–40 Hz ranges. WT mice also have a higher power in the beta band (12–30 Hz) frequency compared to both FX and CON mice (Figure 2D).

### Unit population firing rates show a rescue in oscillation dynamics in CON mice

**Figure 3:**
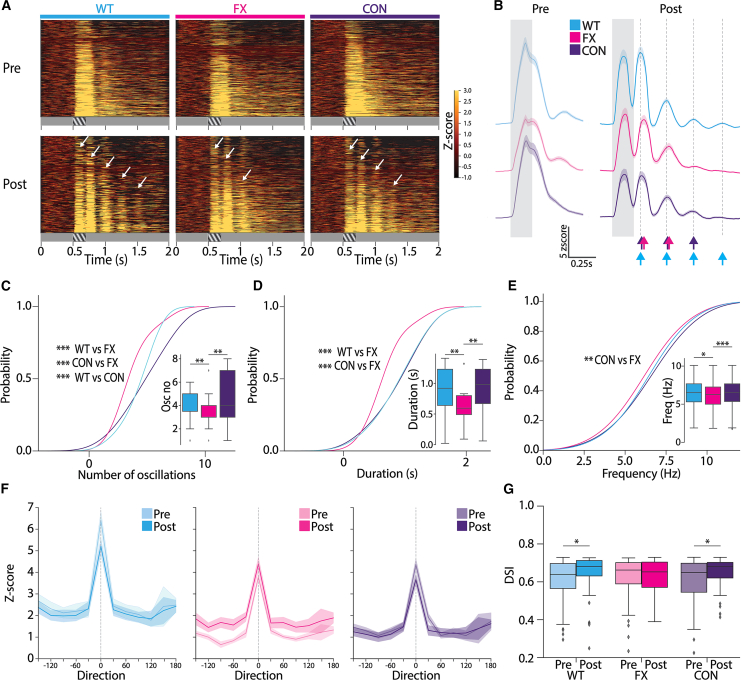
Unit population activity shows a rescue in oscillation dynamics and a restoration of orientation tuning in CON mice (A) Heat maps showing baseline normalized *Z* score firing rates of single units in V1 in WT (cyan), FX (magenta), and CON (indigo) mice, pre- (top; WT: *n* = 563 units, 13 mice, FX: *n* = 554 units, 12 mice, CON: *n* = 592 units, 15 mice) and post- (bottom; WT: *n* = 415 units, 12 mice, FX: *n* = 593 units, 15 mice, CON: *n* = 563 units, 13 mice) visual experience. Also see Figure S1. (B) Baseline normalized *Z* score firing rates averaged across all units shown in (A) pre- (left) and post- (right) visual experience. Arrows indicate peak firing rates in each oscillation cycle for each strain. Also see Figure S1. (C) Cumulative distribution function (CDF) of the number of identified peaks in the baseline averaged *Z* score firing rates post-visual experience shown in (B) for each strain (two-sample Kolmogorov-Smirnov test; WT vs. FX: D = 0.4, *p* = 0.0009, CON vs. FX: D = 0.4, *p* = 0.0003, WT vs. CON: D = 0.3, *p* = 0.01). Inset: Mean number of peaks averaged across mice within each strain post-visual experience (Mann-Whitney U test; WT vs. FX: *p* = 0.0056, CON vs. FX: *p* = 0.0012) (WT: *n* = 47 units post peak detection across 12 mice, FX: *n* = 46 units post peak detection across 15 mice, CON: *n* = 63 units post peak detection across 13 mice). Also see Figure S1. (D) Cumulative distribution function (CDF) of the duration of identified peaks in the baseline averaged *Z* score firing rates post-visual experience shown in (B) for each strain (two-sample Kolmogorov-Smirnov test; WT vs. FX: D = 0.46, *p* = 0.00005, CON vs. FX: D = 0.42, *p* = 0.00007). Inset: mean duration of peaks averaged across mice within each strain post-visual experience (Mann-Whitney U test; WT vs. FX: *p* = 0.005, CON vs. FX: *p* = 0.003) (WT: *n* = 47 units post peak detection across 12 mice, FX: *n* = 46 units post peak detection across 15 mice, CON: *n* = 63 units post peak detection across 13 mice). Also see Figure S1. (E) Cumulative distribution function (CDF) of the frequency of identified peaks in the baseline averaged *Z* score firing rates post-visual experience shown in (B) for each strain (two-sample Kolmogorov-Smirnov test CON vs. FX, D = 0.11, *p* = 0.003). Inset: mean frequency of peaks averaged across mice within each strain post-visual experience (Mann-Whitney U test; WT vs. FX: *p* = 0.031, CON vs. FX: *p* = 0.0008) (WT: *n* = 380 units post peak detection across 12 mice, FX: *n* = 526 units post peak detection across 15 mice, CON: *n* = 508 units post peak detection across 13 mice). Also see Figure S1. (F) Baseline normalized maximum *Z* score firing rates within the stimulus time window (0.5–0.7 s) to 12 different direction (with preferred directions set as 0° for each unit), averaged across units within each strain pre- and post-visual experience (pre: WT: *n* = 115 units, 12 mice, FX: *n* = 162 units, 15 mice, CON: *n* = 196 units, 13 mice; post: WT: *n* = 86 units, 12 mice, FX: *n* = 153 units, 15 mice, CON: *n* = 215 units, 13 mice). (G) Direction selectivity index was calculated from baseline normalized *Z* score firing rates during stimulus presentation (0.5–0.7 s) to 12 different orientations, averaged across units showing direction preference, pre-, and post-visual experience. (Mann-Whitney U test; WT: pre vs. post, *p* = 0.026, CON: pre vs. post: *p* = 0.027) (pre: WT: *n* = 115 units, 12 mice, FX: *n* = 162 units, 15 mice, CON: *n* = 196 units, 13 mice; post: WT: *n* = 86 units, 12 mice, FX: *n* = 153 units, 15 mice, CON: *n* = 215 units, 13 mice). Also see Figures S1–S3. ∗All statistics done on *n*, number of units. Data are shown as boxplots with 25th percentile, median, and 75th percentiles, and whiskers indicate min and max values. ∗*p* < 0.05, ∗∗*p* < 0.01, ∗∗∗*p* < 0.001.

In order to characterize the neuronal firing rates in V1, we analyzed the unit population firing rates pre- and post-visual experience. Baseline (0–0.5 s)-normalized unit z-scores were averaged across trials and mice within each strain. Post-visual experience *Z* score firing rates show persistent, stimulus-locked, low-frequency theta oscillations similar to LFP activity (Figures 3A–3E and S1). We further characterized the dynamic features of these oscillations by employing a peak detection function to identify synchronized firing rate patterns. We found that CON mice show a higher mean number of oscillation cycles compared to FX mice (Figures 3C and S1). Surprisingly, the probability density function of the identified peaks shows that the oscillation number distribution across all units is higher in CON mice compared to both FX and WT mice (Figure 3C). We then calculated the mean duration of these oscillations, which was also significantly longer in CON mice compared to FX (Figures 3D and S1). Frequency analysis of the detected peaks revealed a significant shift toward a higher range in CON mice, similar to WT, while FX oscillates at a lower frequency (Figures 3E and S1). In summary, CON mice have a higher number of oscillation cycles, longer duration and oscillate at a higher frequency compared to FX mice (Figures 3C–3E and S1). Additionally, we separated the identified units as putative fast-spiking (FS) or regular-spiking (RS) based on unit waveforms (Figures S2 and S3). We see that FX mice have a significantly lower percentage of active FS neurons post-visual training compared to CON mice, which is not seen prior to visual familiarity (Figure S2). This percentage is lower compared to WT mice but does not reach significance. Here, we see that FX mice have decreased functionally active FS neurons, of which PV+ neurons form a large subset, which is rescued in the CON strain. Furthermore, we also separated FS and RS units across cortical layers of V1 (Figure S3). Overall, we see no significant differences in unit *Z* score firing rates in L1 and L6 across all three strains. In L2/3, RS units of WT and CON mice have longer duration of oscillations compared to FX, while this is reversed in FS units, where FX has longer durations compared to WT and CON. In L4, we see that WT RS units oscillate longer compared to both FX and CON strains. In L5, both RS and FS units have longer durations compared to FX in both WT and CON mice. L5 receives strong feedback from higher visual areas (HVAs) post-visual experience, which drives the changes forming the recurrent oscillatory network.^18^^,^^20^^,^^25^ Post-synaptic restoration of FMRP in PV+ interneurons might have a rescuing effect on L5–L4 RS synaptic strengthening upon visual training. However, the number of RS and FS units separated by layer is significantly lower, and it is hard to draw any clear conclusions.

Previously, we have extensively characterized the attenuated visual familiarity response in FX.^20^^,^^21^ Here, we see that restoration of FMRP in PV+ interneurons rescues this attenuated response, restoring the resonance frequency, duration, and partially the strength of the visual familiarity evoked oscillations.

### CON mice show improved direction tuning post-visual experience

Tuning properties are a strong indicator of perceptual adaptation and stimulus selectivity dependent on the inhibitory activity of interneurons, which are impaired and broader in FX mice.^4^^,^^26^^,^^27^ Previously, we found that passive visual experience sharpens tuning and increases direction selectivity in WT mice.^18^ To study how restoring function in PV+ interneurons affects direction tuning in FX mice, we analyzed *Z* score firing rate responses of the unit population to 12 different directions of sinusoidal grating stimulus across all three strains. We recorded from mice both pre- and post-visual experience. During recordings, mice were shown 20 trials of 12 different directions of sinusoidal grating stimuli (0.04 cycles/degree) presented pseudo-randomly. We looked at the baseline normalized average *Z* score of firing rates within the stimulus time window for each of the 12 directions and assigned a preference to orientation for each unit with the maximum *Z* score response to a given orientation. The preferred direction was plotted as 0°, and all other directions were re-aligned with respect to that. We then plotted the baseline normalized z-scores averaged across mice within each strain with respect to the preferred direction (Figure 3F). The overall z-scores were lower for all directions post-training compared to pre-training in WT and CON mice (Figure 3F). This is consistent with research that shows passive visual experience results in a lowering of firing rates in WT mice.^18^^,^^28^ However, here, we see that FX mice have higher firing rates post-visual experience compared to pre-training (Figure 3F). We then quantified the direction selectivity index (DSI) across all neurons that showed a preference, across strains. Visual experience resulted in an increase in DSI in WT mice, which was also seen in the CON strain (Figure 3G). However, FX mice show no significant change in DSI post-training (Figure 3G). These results show that in WT mice, visual experience sharpens direction tuning curves and reduces overall firing rates. In contrast, FX mice show broader tuning curves and higher firing rates post-visual experience, indicating a decrease in tuning. These results show that restoring FMRP expression in PV+ interneurons rescues tuning curve sharpening and normalizes firing rates across directions post-experience, resembling WT mice.

### CON mice perform better in a visual discrimination task compared to FX mice

**Figure 4:**
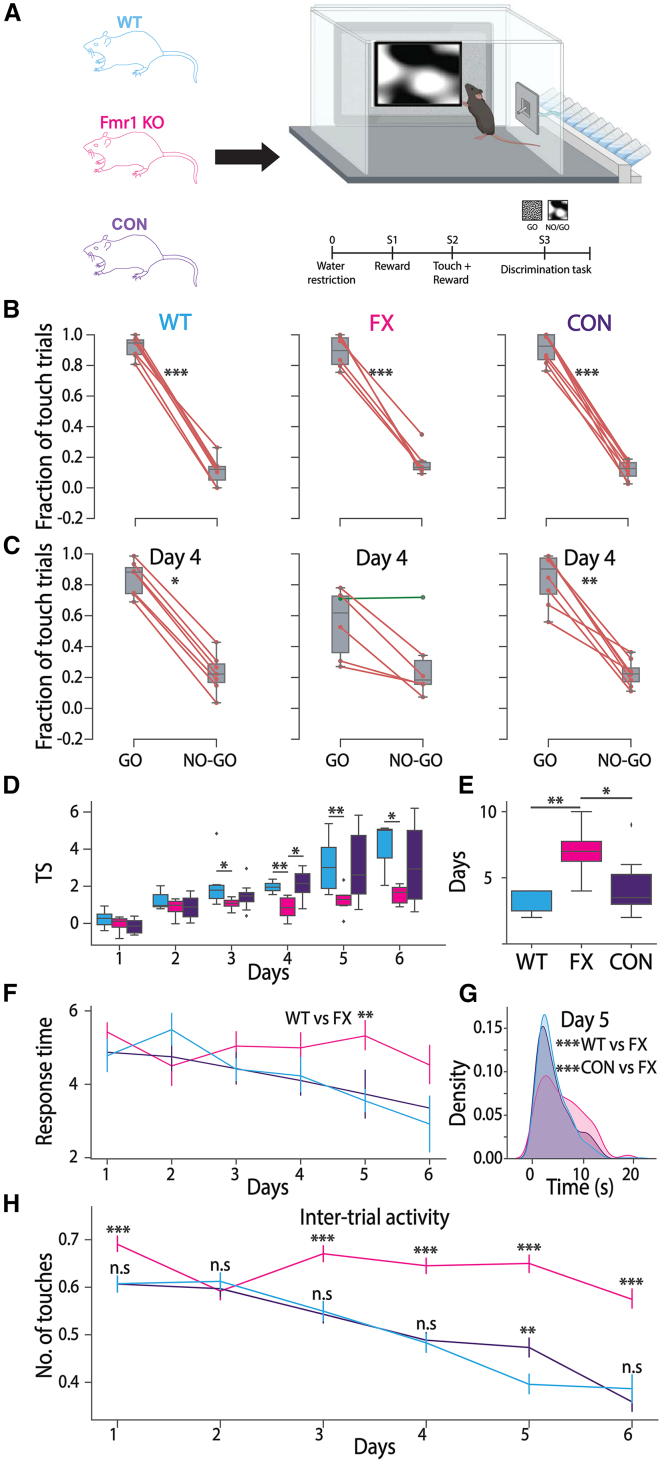
CON mice perform better than FX mice in a Go/No-Go visual discrimination task (A) Schematic showing the operant conditioning paradigm used for the visual discrimination task using freely moving touchscreen chambers. Created in BioRender. Cheng, X. (2025) https://BioRender.com/dt8yp2w. (B) Paired boxplots showing the percentage of touches for Go and No-Go trials, on the last day of training for each strain (Wilcoxon signed-rank test; WT: *p* = 2.55e-11; FX: *p* = 1.09e-09; CON: *p* = 7.45e-07). (C) Paired boxplots showing the percentage of touches for Go and No-Go trials for each strain and on day 4 of training (Wilcoxon signed-rank test; WT: *p* = 0.016; CON: *p* = 0.008) for WT (cyan, *n* = 7 mice), FX (magenta, *n* = 6 mice), and CON (indigo, *n* = 8 mice) mice. (D) Training scores averaged across mice within each strain for each day over the period of training in WT (cyan, *n* = 7 mice), FX (magenta, *n* = 6 mice), and CON (indigo, *n* = 8 mice) mice (Mann-Whitney U test; day 5: WT vs. FX, *p* = 0.005) (Mann-Whitney U test; day 3: WT vs. FX, *p* = 0.022; day 4: WT vs. FX, *p* = 0.0012, CON vs. FX, *p* = 0.02; day 5: WT vs. FX, *p* = 0.008; day 6: WT vs. FX, *p* = 0.048). (E) Total number of days taken to achieve a training score of 2, averaged across mice within each strain, WT (cyan, *n* = 7 mice), FX (magenta, *n* = 6 mice), and CON (indigo, *n* = 8 mice) mice (Mann-Whitney U test; WT vs. FX, *p* = 0.006, CON vs. FX, *p* = 0.044). (F) Median response times across mice within each strain for each day over the period of training in WT (cyan, *n* = 7 mice), FX (magenta, *n* = 6 mice), and CON (indigo, *n* = 8 mice) mice. (G) Probability density functions showing response times on day 5 of training in WT (cyan, *n* = 7 mice), FX (magenta, *n* = 6 mice), and CON (indigo, *n* = 8 mice) mice (Kolmogorov-Smirnov test; WT vs. FX: *p* = 2.4e-10, CON vs. FX: *p* = 2.7e-07). (H) Total number of touches in between trials (during the absence of visual stimulus presentation) for each day over the period of training, averaged across mice within each strain, WT (cyan, *n* = 7 mice), FX (magenta, *n* = 6 mice), and CON (indigo, *n* = 8 mice) mice (Mann-Whitney U test; WT vs. FX; day 1: p = 4e-4, day 3: *p* = 3.3e-6, day 4: *p* = 2.6e-10, day 5: *p* = 3.7e-19, day 6: *p* = 9.3e-7, WT vs. CON; day 5: *p* = 0.007, CON vs. FX; day 1: *p* = 9.9e-5, day 3: *p* = 4.5e-7, day 4: *p* = 4.4e-10, day 5: *p* = 7.2e-11, day 6: p = 8e-13). Data are shown as boxplots with 25th, median, and 75th percentiles, and whiskers indicating min and max values. ∗*p* < 0.05, ∗∗*p* < 0.01, ∗∗∗*p* < 0.001.

To ascertain if the partial rescue of visual familiarity encoding in CON mice could have a direct impact on active learning behavior, we used an operant conditioning paradigm and analyzed the performance of mice across all three strains. Mice were placed under water restriction and trained in a Go/No-Go visual discrimination task in visual touchscreen chambers.^29^ Filtered pink noise patterns with two different spatial frequencies were used as the Go and No-Go stimuli. Mice were given a water reward if they touched the screen during the Go stimulus presentation (0.12 cycles/degree spatial frequency, 0.75 Hz temporal frequency, 3 s) and given a time-out punishment if they touched the screen during the No-Go stimulus presentation (0.03 cycles/degree sf, 0.75 Hz tf, 3 s). Mice were gradually trained in stages where they learned to recognize the water port for reward (Stage 1), touch a gray screen to receive reward (Stage 2), and finally discriminate between the visual stimuli to receive reward for the Go stimulus (Stage 3) (Figure 4A). TSs were calculated using normalized correct touches to Go trials (Hits [H]) and incorrect touches to No-Go trials (false alarms [FA]). We discovered that all three strains of mice were able to eventually discriminate between the two stimuli on stage 3, reaching a TS greater than 2, which we considered to be “expert” at the task (Figures 4B and E). However, FX mice take a significantly longer number of days to reach this threshold compared to WT mice (Figures 4B–4E). Interestingly, CON mice take fewer days than FX but are not as quick as the WT (Figures 4B–4E). Further, we analyzed the time taken by mice to touch the screen once the stimulus was presented for Go trials as an indicator of response time. Gradually, as the mice learned the task, response times decreased, indicating familiarity with the task and quicker decision-making. WT and CON mice showed quicker response times on day 5, compared to FX mice, which were slower to respond, though they did start to respond quicker as training progressed (Figures 4F and 4G). Additionally, we quantified inter-trial touch activity as a metric for hyperactive behavior (Figure 4H). Here, we see a drastic phenotype, wherein FX mice touch the screen between trials, while no visual stimulus is present, a significantly higher number of times compared to both WT and CON mice. This hyperactive behavior persisted throughout the training period, even after the mice had learned the task.

### CON mice preferentially interact with mice over object

**Figure 5:**
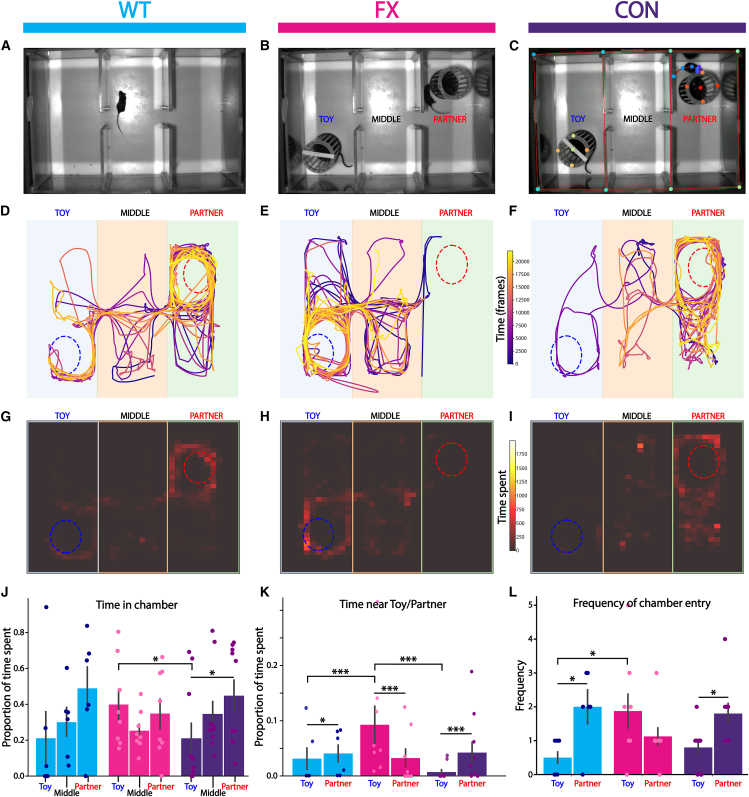
Social interaction behavior in the three-chamber test (A) Image showing mouse habituation to the three chamber social test rig. (B) Image showing mouse interaction in the three chamber social test rig. (C) Image showing mouse tracking using DeepLabCut software. (D) Example mouse movement trajectories with color inidicating time by frames for WT mice. (E) Same as (D) but for FX mice. (F) Same as (D) but for CON mice. CON (*n* = 10 mice), FX (*n* = 8 mice), WT (*n* = 6 mice). (G) Heatmaps showing time spent in binned regions of the chambers for an example WT mouse. (H) Same as (G) but for FX. (I) Same as (G) but for CON. (J) Proportion of time spent of the total time (10 min) in each chamber for WT (cyan, *n* = 6 mice), FX (magenta, *n* = 8 mice), and CON (indigo, *n* = 10 mice) mice. Mann-Whitney U test; WT: toy vs. partner; *p* = 0.009, WT: partner vs. middle; *p* = 0.009, FX: toy vs. middle; p = 6e-4, FX: partner vs. middle; *p* = 0.01, CON: partner vs. middle; *p* = 0.007. (K) Proportion of time spent of the first minute within a small area limited to either the toy cage or the partner mouse cage, for WT (cyan, *n* = 6 mice), FX (magenta, *n* = 8 mice), and CON (indigo, *n* = 10 mice) mice. Mann-Whitney U test; WT: toy vs. partner; *p* = 0.012, FX: toy vs. partner; *p* = 8.2e-10, CON: toy vs. partner; *p* = 6e-11, WT vs. FX; within toy: *p* = 2.4e-7, CON vs. FX; within toy: *p* = 1.1e-26. (L) Frequency of entry for the first minute into the partner mouse chamber and the toy mouse chamber for WT (cyan, *n* = 6 mice), FX (magenta, *n* = 8 mice), and CON (indigo, *n* = 10 mice) mice. Mann-Whitney U test; WT: toy vs. partner; *p* = 0.031, CON: toy vs. partner; *p* = 0.01, WT vs. FX; within toy: *p* = 0.048. Data are presented as mean ± SEM. ∗*p* < 0.05, ∗∗*p* < 0.01, ∗∗∗*p* < 0.001.

Apart from learning deficits, there are a number of behavioral phenotypes associated with FX, such as hyperactivity, repetitive behaviors, anxiety, and social impairments. Here we see hyperactive behavior in our open-field task in FX mice, which is rescued in the CON strain. Additionally, in order to study social behavior in our conditional restoration strain, we utilized the three chamber test (Figure 5A). Using this test, we were able to analyze interaction behaviors for each strain with a partner mouse (WT strain) and a toy mouse object. All partner mice were habituated to the setup for 10 min each day over 3 days. For testing, each test mouse was allowed to roam free in the three-chamber setup for 10 min for habituation. Following this, the mouse was restricted to the middle chamber, while a toy and partner mouse were placed in the other two chambers (Figure 5B). The mouse was then allowed to roam freely for 10 min, while we recorded behavior. Using DeepLabCut software,^30^^,^^31^ we tracked mice movements and analyzed behavioral patterns for the first minute of the recording (Figures 5C–5I). We see that the proportion of time spent in each chamber is similar across all strains; however, FX mice spend more time in the toy chamber compared to CON mice (Figure 5J). CON mice also spend a significantly larger amount of time in the partner mouse chamber compared to the toy chamber. We then analyzed the amount of time spent within a small region around the toy/partner for each mouse. Here we see that WT and CON mice spend more time interacting with the partner mouse than the toy object (Figure 5K). This is flipped in FX mice, who prefer to spend more time around the toy than the partner mouse. FX mice spend a significantly larger portion of time near the toy object compared to both WT and CON strains. We also observe that both WT and CON mice enter the partner chamber more frequently than the toy chamber, while FX mice show no significant difference (Figure 5L). FX mice enter the toy chamber a significantly larger number of times compared to WT. Overall, from these results, we see that FX mice preferentially interact with an object over a partner mouse, consistent with the literature indicating social impairments. The CON strain shows social interaction similar to the WT group, preferring the partner mouse over the toy. Restoration of FMRP in PV+ interneuron in other brain regions, such as the amygdala and the anterior cingulate cortex (ACC), typically associated with social behaviors, could explain the improvement seen in the CON strain.

## Discussion

Studies have shown that low-frequency oscillations in cortical regions are associated with memory encoding.^2^ Visual familiarity evokes low-frequency 4–8 Hz theta oscillations in V1 of mice, which are attenuated in FX mice with lower frequency, amplitude, and power.^20^ This attenuation is associated with impaired connectivity across cortical layers within V1, notably a loss in strengthening of L5 RS projections onto layer L4 FS interneurons post-visual experience.^20^ Here, we have shown that conditionally restoring FMRP expression in PV+ interneurons, which make up the largest percentage of FS interneurons, rescues several critical impairments of familiarity-evoked oscillations in FX. LFP activity post-visual experience shows significantly lower VEP amplitudes in FX mice compared to WT mice, whereas CON mice do not differ significantly from WT. Though the VEP amplitudes in CON mice are not significantly higher than FX, these results indicate an intermediate phenotype or a partial rescue. Power spectral analysis in the theta (4–8 Hz) frequency band shows reduced power in FX mice compared to WT, while CON mice are not significantly different from WT. Here, too, we see that while the power is higher in CON mice compared to FX mice, it is not significant. This type of partial rescue in LFP activity might be due to improvements in activity dependent strengthening of PV+ synapses but not in other synapses of the oscillatory circuit. Neuronal population activity shows a stronger rescue in CON mice, showing significant improvements in V1 familiarity response compared to FX. Population-averaged Z-scored firing rates in CON mice have an increased number of oscillatory cycles, higher oscillation frequencies, and increased oscillation durations compared to FX mice, suggesting substantial rescue of V1 network dynamics.

We also analyzed direction tuning properties before and after visual experience. Previous studies have demonstrated that tuning in pyramidal cells is regulated by FS interneurons, and their dysfunction leads to degraded tuning in FX mice.^4^^,^^13^^,^^26^^,^^27^ Consistent with these findings, our results also show impaired tuning in FX mice. While WT mice show sharpening in direction tuning curves and overall reduced firing rates following visual experience, FX mice do not show sharpening and, in fact, demonstrate higher firing rates post-visual experience. Notably, the CON mice show sharpening and decreased firing rates similar to WT mice. Furthermore, the DSI is significantly higher post-experience in both WT and CON mice, but this increase is absent in FX mice.

FX has been associated with impairments in the inhibitory network, attributed to reduced number, diminished function, and structural and morphological aberrations of PV+ interneurons.^4^^,^^8^^,^^12^^,^^13^ Furthermore, studies show that restoring PV+ interneuron function via pharmacological, chemogenetic, and optogenetic interventions improves circuit-level sensory responses and learning impairments in FX mice.^4^^,^^9^^,^^12^^,^^13^ However, impairments in the excitatory network are equally important. Conditional knockout of FMRP in both excitatory and inhibitory neurons has been shown to result in FX-like phenotypes.^16^^,^^32^^,^^33^ FMRP negatively regulates the expression of several proteins that are important in development and synaptic function. This regulation is drastically dependent on neuron subtype and brain region, which manifests in a range of cell-level and local circuit aberrations in KO conditions. *Fmr1* KO models show a decrease in local excitatory drive, specifically onto L4 FS interneurons,^20^^,^^34^ and interestingly, knockouts in excitatory neurons alone result in altered structure and function of PV interneurons.^32^ Furthermore, conditional re-expression of FMRP in excitatory neurons improved cortical circuit impairments.^35^ This suggests that activity dependent plasticity changes in the network can be rescued through both pre- and postsynaptic reintroduction of FMRP. FMRP dysregulation, although cell specific, can have drastic effects on the local circuit, and consequently, cell-targeted restorative efforts can improve the local network as a whole.

Within PV+ cells, FMRP expression can have an effect through several pathways, including the expression and organization of ion channels that determine intrinsic cell excitability properties. The theta frequency resonance of neocortical L5 pyramidal neurons driven by optogenetic stimulation of PV+ interneurons is dependent on hyperpolarization-activated cyclic nucleotide-gated channel 1 (HCN 1).^36^ In general, FX mice show varied, either high or low, modulation of HCN channels depending on the brain region.^37^^,^^38^^,^^39^ Studies have shown that FMRP directly interacts with HCN channels in a cell autonomous way and modulates hyperpolarizing currents (I_h_) by regulating functional HCN channels on dendrites. Notably, both hypo- and hyper-functionality of HCN channels in different brain regions was rescued by FMRP reintroduction, restoring dendritic function.^40^ HCN 1 channels in presynaptic terminals of PV+ interneurons enhance GABAergic transmission onto CA1 pyramidal cells.^41^ In our results, we see a rescue in the frequency and duration of familiarity-evoked theta oscillations, which could be due to FMRP restoration of HCN-mediated inhibitory control. In another ion channel pathway, FMRP regulates experience dependent expression of Kv3.1 channels, which are responsible for fast-spiking in PV+ interneurons.^42^^,^^43^^,^^44^ Hypo-function of PV+ interneurons observed in FX was ameliorated upon pharmacological treatment with an allosteric modulator of the Kv3.1 channel, improving coupling with excitatory neurons, and decreasing hypersensitivity.^12^ In our CON restoration strain, we see an increase in functionally active PV+ cells after visual experience when compared to FX. This might be explained by a similar mechanism of rescue in experience-dependent expression of Kv3.1 channels, postsynaptically enhancing excitatory cell coupling. Impairments in this coupling can explain the loss of strengthening of the V1 L5 RS-L4 FS recurrent pathway in FX after visual experience,^20^ a rescue of which might contribute to the improved oscillatory response in the CON strain.

Our results indicate that conditional restoration of FMRP in PV+ interneurons is sufficient to rescue multiple aspects of the visual familiarity-induced oscillations in V1, likely due to the increased function of PV+ interneurons leading to improved circuit-level synchronization and activity.

Finally, we also characterized behavioral performance across the three strains using a visual discrimination task. Consistent with previous reports, FX mice demonstrated learning impairments and performed poorly in behavioral tasks compared to WT mice.^4^ FX mice took longer to reach a TS score of 2 compared to WT mice. CON mice achieved this TS score significantly faster than FX mice. Analysis of the response times during the Go/No-Go visual discrimination task further demonstrated that both WT and CON mice responded faster after a few days of training, compared to FX mice, while they eventually started to respond quickly as well.

We also see hyperactive behavior in FX, with mice showing a high number of inter-trial touches in the open field task. Furthermore, the three-chamber social behavior test showed social interaction impairments in FX mice, preferring to interact with a toy object over a partner mouse. CON mice preferentially interacted with the partner mouse, indicating a rescue in social interaction impairments. Social and anxiety-related behaviors are strongly associated with dysregulation in the GABAergic system in brain regions such as the amygdala, the ACC, and the cerebellum. Restored PV+ network in these brain regions of the CON strain completely rescues these behaviors.

Our findings demonstrate that restoring FMRP expression in PV+ interneurons rescues not only the network-level oscillatory responses linked to visual familiarity but also improves learning and behavioral performance. Together, this suggests potential circuit-specific therapeutic approaches to target cognitive deficits associated with FX and ASD.

### Limitations of the study

This study is limited by the fact that all electrophysiology recordings were limited to V1. To fully understand the restorative effects of the CON strain, it is important to characterize network activity in other brain regions, especially in regions associated with behavioral phenotypes, of which we see a rescue here. Furthermore, future studies recording V1 activity during a visual discrimination task will be valuable to better understand circuit-level changes leading to learning improvements in the CON strain. It would also be beneficial to study cellular-level changes of membrane properties, excitability, and structure/morphology in PV+ interneurons of the CON strain using patch clamp techniques. Post-visual training, synaptic changes across the cortical layers of V1, forming the oscillatory circuit, can be studied using channelrhodopsin-2-assisted circuit mapping and would shed light on the underlying mechanism of FMRP rescue of the network. We also note that the PV promoter is active past the critical developmental stage of the brain, and hence, any developmental impairments seen in *Fmr1* KO models are expected to exist in the CON strain. Models having full restoration during the developmental phase might be of interest to compare rescue effects in the current CON strain in future studies.

## Resource availability

### Lead contact

Further information and requests for resources and reagents should be directed to and will be fulfilled by the lead contact, Alexander A. Chubykin (chubykin@purdue.edu).

### Materials availability

This study did not generate new unique reagents.

### Data and code availability

• All data reported in this paper are available from the lead contact upon request.
• This paper does not report original code.
• Any additional information required to reanalyze the data reported in this paper is available from the lead contact upon request.

## Acknowledgments

The authors thank all Chubykin lab members for their feedback and support during the development of the project, specifically, Michael Zimmerman, Mang Gao, and Yu Tang. The authors also thank Dr. Sotiris Masmanidis for the provided 64-channel silicon probes. This work was funded by the 10.13039/100000002National Institutes of Health (NIH) under grant no. R01 MH116500.

## Author contributions

S.N. and A.A.C. conceptualized and designed the experiments. S.N., V.S., P.A.E., and M.F. performed experiments. S.N. performed all data analyses. S.N and X.H performed data analysis for the three-chamber test. All authors reviewed and edited the manuscript.

## Declaration of interests

The authors declare no competing interests.

## STAR★Methods

### Key resources table

**Table:** 

REAGENT or RESOURCE	SOURCE	IDENTIFIER
**Chemicals**		

Monosodium Phosphate	Santa Cruz	sc-202342
Sodium Bicarbonate	DOT scientific, Inc	DSS22060
Dextrose	DOT scientific, Inc	7203B
Sodium Chloride	DOT scientific, Inc	DSS23020
Calcium Chloride	DOT scientific, Inc	DSC20010
Magnesium Chloride	DOT scientific, Inc	DSM24000
Potassium Chloride	DOT scientific, Inc	DSP41000

**Experimental Models: Organisms/Strains**		

C57BL/6	The Jackson Laboratory	C57BL/6
Fragile X knock-out (Fmr1 KO)	The Jackson Laboratory	B6.129P2-Fmr1tm1Cgr/J, JAX Stock No. 003025
Fmr1cON	Provided by David Nelson	Fmr1cON provided by David Nelson
PV-Cre	The Jackson Laboratory	B6.129P2-Pvalbtm1(Cre)Arbr/J: JAX Stock No. 008069

**Antibodies**		

anti-Parvalbumin	Bosterbio	Cat# M04041
7G1 anti-FMRP	DSHB	RRID: AB_528251
Alexa Fluor 488 goat anti-mouse IgG (H + L)	ThermoFisher	RRID: AB_2534069
Alexa Fluor 647 goat anti-chicken IgG (H + L)	ThermoFisher	RRID: AB_2535866

**Software and algorithms**		

Python	Python 3.8.8	https://www.python.org/
Anaconda Distribution	Anaconda Inc.	Anaconda for Python 3
MATLAB	Mathworks	https://www.mathworks.com
Kilosort 1.5	Kilosort	https://github.com/MouseLand/Kilosort
ABETT II	Lafayette Instruments	https://lafayettelifesciences.com/
DeepLabCut	DeepLabCut	RRID: SCR_021391; https://github.com/AlexEMG/DeepLabCut

**Other**		

C&B-Metabond	Parkell	S380
Animal Temperature Controller	World Precision Instruments (WPI)	ATC-2000
Motorized Stereotax	Neurostar	Single robot stereotax
Isoflurane Vaporizer	Parkland Scientific	V3000PK
Micromanipulator	Scientifica	PatchStar Micromanipulator with PatchPad
Stereo Microscope	AmScope	SM-4TZ-144A
64 Channel Silicon Probe	Masmanidis Lab, UCLA	64D
Acquisition Board	OpenEphys	Acquisition Board
128 Channel Amplifier Board	Intan Technologies	RHD2000
Arduino Board	Arduino	A000066
I/O Board	OpenEphys	I/O Board
Electroplating Board	Intan Technologies	RHD2000 Electroplating Board
Interface Cable	Intan Technologies	RHD2000 SPI interface Cable
Touchscreen Chamber	Lafayette Instrument	Model 80614A
Vibratome	Leica	VT1000

### Experimental model and study participant details

#### Animals

All experimental procedures were approved by the Purdue Animal Care and Use Committee (PACUC, protocol number 140800111). All mice were subjected to a standard 12-h light and 12-h dark circadian cycle. Three-to four-month-old homozygous male *Fmr1* KO (*B6.129P2*-*Fmr1tm1Cgr/J*, JAX Stock No. 003025), B6 (*C57BL/6*, JAX), and *Fmr1*cON/PV-Cre (*Fmr1*cON provided by David Nelson, crossed with *B6.129P2-Pvalbtm1(Cre)Arbr/J*: JAX Stock No. 008069) were used for the experiment. The genotypes were blinded for all experiments during the data collection.

### Method details

#### Immunohistochemistry

Mice (WT, FX, and *Fmr1*cON/PV-Cre) were injected with 500 nL of AAV hSyn DOI mCherry in V1 (AP: 0.8 mm, ML: 3 mm, from lambda; 250 nL at DV: 700 μm, and 250 nL at DV: 400 μm). Perfusions were performed after two weeks from injections. Whole brains from perfusions were stored in PFA at 4°C overnight. Brain slices of 100 μm thickness were made in 1X PBS using a vibratome. Brain slices were washed in washing buffer (1% Tween 20 in 1X PBS) for 15 min on a shaker at RT, repeated 3 times. Slices were treated with Sodium Citrate solution (10 mM) for 45min at 75°C in a water bath. They were gradually cooled to RT over 30 min. Slices were washed 3 times for 15 min in the washing buffer. Slices were then treated with blocking solution (5% BSA in 1% Tween 20 1X PBS) for 90 min on a shaker at RT. Slices were incubated in anti-Parvalbumin primary antibody (Bosterbio, cat# m04041) diluted to 1:1250 in blocking solution at RT for 2 h. Slices were then incubated in 7G1 anti-FMRP primary antibody (DSHB) diluted 1:20 in blocking solution, at 4°C overnight. Slices were washed 3 times in washing buffer for 15 min on a shaker at RT. They were then incubated on a shaker for 2 h at RT with Alexa Fluor 488 goat anti-mouse IgG (H + L) and Alexa Fluor 647 goat anti-chicken IgG (H + L) secondary antibodies, diluted 1:1200 times in 1X PBS with 0.1% Tween 20. Slices were washed 3 times for 15 min in washing buffer on a shaker at RT. Slices were incubated with DAPI (0.1 mg/mL) diluted 1:100 times in 1X PBS for 10 min at RT on a shaker. Slices were washed in washing buffer 3 times for 10 min at RT on a shaker. Slides were made by placing the brain slices in mounting media (90% Glycerol; 10% 1X PBS). Coverslips were air-sealed. Samples were imaged using a confocal microscope. Images were acquired in a single optical plane using identical acquisition settings.

#### Headplate installation surgeries

All surgeries were performed on a stereotaxic frame (Neurostar) while mice were anesthetized using 1.5–2% isoflurane gas delivered through a nose cone (SomnoSuite system). Mice were placed on a heating pad throughout the surgery. Ocular lube was applied to their eyes to prevent drying. The scalp was removed using heat-sterilized scissors. Connective tissue was removed from the surface of the skull using 3% hydrogen peroxide. Using a scalpel, scratches were made on the surface of the skull in a checkerboard pattern to ensure strong fixation of the headplate. The primary visual cortex (V1) was marked on the skull with a permanent marker using stereotaxic coordinates (with respect to lambda: ±3.0 mm lateral, 0.5 mm anterior). A gold-plated grounding pin (WPI 5482) was attached to the skull with the tungsten wire sitting above the brain surface. A customized head plate was then placed on the surface of the brain. The grounding pin and the headplate were then sealed to the skull using Metabond (Parkell S380). Mice were allowed to recover on the heat pad before being transferred to the cage. For three days post-surgery, Carprofen (Rimadyl, 0.01 mL/g of 0.05 mg/mL for each mouse) and enrofloxacin (Baytril, 0.005 mL/g of 1 mg/mL for each mouse) were injected subcutaneously into the mice. Mice were started on habituation after three days to allow for recovery.

#### Passive visual stimulus experience paradigm

Head-fixed mice were habituated to the visual stimulus set up for 3–4 days, during which they were placed in front of a monitor displaying a gray screen for 60 min each day. Mice were then trained to a 200 ms sinusoidal grating stimulus (60° orientation, 0.04 cycles/degree) interspersed with a gray screen shown 200 times each day for 5–6 days. For direction tuning, sinusoidal gratings of 12 different durations were generated by rotating the grating stimulus by increments of 30°. All visual stimuli for passive visual experience were presented using the PsychoPy software. The 60° grating stimulus, interspersed with a gray screen, was presented 20 times during recording sessions, while the 12 different directions were presented 20 times each in a pseudorandom sequence, interspersed with a gray screen for direction tuning recordings. For the visual discrimination task, filtered pink noise images of two different spatial frequencies (0.12, 0.03 cycles/degree) were generated using MATLAB. The images were then used to generate movies on Python where the light intensities of each pixel changed with a sinusoidal function of time at a 0.75 temporal frequency. The movies were then presented in the touchscreen chambers using ABETT code software.

#### Extracellular recordings and data acquisition

*In vivo* extracellular recordings were performed on mice using 64-channel silicon probes inserted normally into the primary visual cortex (V1). Craniotomies were made over V1 using the stereotaxic frame. Artificial cerebrospinal fluid was added over the craniotomy (ACSF; composition in mM: 124 NaCl, 26 NaHCO_3_, 10 Dextrose, 2.5 KCl, 2 CaCl_2_, 1.25 NaH_2_PO_4_, 0.8 MgCl_2_; pH = 7.3–7.4), after which the probe was placed on the surface of V1 using the NewScale manipulator. The probe was inserted into V1 at a rate of 50 μm/min to a depth of 950 μm. Data acquisition was started 15 min after insertion using OpenEphys acquisition hardware and software at 20 kHz.^17^ For LFP analysis, raw data was passed through 300 Hz low-pass filter and down-sampled to 1 kHz. For unit analysis raw data was band-pass filtered between 300 and 6000 Hz and spikes were clustered using Kilosort. Phy GUI was used to manually inspect all units and remove noisy units.

#### Operant conditioning behavior paradigm

Mice were placed on water restriction to train them in the operant conditioning paradigm. Mice weights were recorded one day before placing them on water restriction and used as their baseline weight. Mice were weighed every day after that and given a minimum of 1 mL of water per day. Once mice reached 80% of their baseline weight, they were started on the training paradigm. Mice were trained in multiple stages before completing the final visual discrimination task to acclimatize to the training chamber. Mice were first placed in the freely moving touchscreen chamber (Lafayette instrument) on stage 1 where they received water (40 μL) from the water port, signaled by a light turning on near the port, in intervals of 5 s. Once they learned to drink the water from the port for at least 70 trials, mice were moved to the next stage. In stage 2, mice had to touch a gray screen displayed on the touchscreen to receive a water reward. Once they completed 80% of trials correctly to receive water, mice were moved to the next stage, the visual discrimination task. On stage 3, or the visual discrimination task, mice were either shown a Go stimulus (filtered pink noise 0.12 sf, 0.75 tf) or No-Go stimulus (filtered pink noise 0.03 sf, 0.75 tf) for 3 s in a pseudo-random order interspersed with a 5-s gray screen between trials. Mice were given a water reward if they touched the screen during the Go stimulus and a 10-s timeout punishment if they touched the screen during the No-Go stimulus.

#### Three chamber social behavior test

The three chamber set up was made available by the Purdue Animal Behavior Core facility. Partner mice were habituated to the three chamber setup for 10 min each day over 3 days. On the day of the social interaction test, the mouse to be tested was placed in the middle chamber and allowed to roam freely in all three chambers for 10 min. After that, the mouse was moved to the middle chamber and the doors to the other two chambers were closed. A partner mouse was placed in a cylindrical cage with open bars in either the left or right chamber, at the top or the bottom, in a random way. Similarly, a toy mouse was placed in an identical cage and placed in the opposite chamber/position of the partner mouse. The doors to both chambers were released, and the mouse was allowed to roam freely for 10 min. The entire session was recorded using IR light cameras with Noldus Media Recorders. All partner mice were ensured to be the same gender as the mice being tested. Test days were performed at the same time of day across all three strains.

### Quantification and statistical analysis

#### Immunohistochemistry analysis

All images were analyzed blindly in a semi-automated workflow with FIJI. PV-positive cells were segmented by applying a fixed fluorescence threshold to the PV channel, followed by mask creation, hole filling, and particle detection to obtain individual PV ROIs. For each ROI, mean fluorescence intensity was measured in the FMRP channel, and cells were classified as PV+ FMRP if the intensity exceeded a predefined threshold. Separately, the FMRP channel was thresholded and segmented to count all FMRP-positive cells. This allowed classification of cells as PV only, FMRP only, or PV+ FMRP. Counts were exported automatically as CSV files and plotted using Python. Mann-Whitney U test was used for non-normal distributions. All statistical details can be found in the figure legends.

#### *In vivo* electrophysiology data analysis

Data analysis was performed using Python code written in our lab. Raw data was acquired at a rate of 20 kHz and passed through a 300 Hz low bandpass filter. The data was then down-sampled to 1 kHz, and a 60 Hz notch filter was used to remove noise. For LFP traces, the channel with the strongest visual response (0.5–0.7 s) was selected for each mouse and averaged across the 20 trials. Comparisons were made across groups by averaging the LFPs for all mice within each strain. VEP analysis was done by identifying time windows for each cycle from averaged LFP traces, and VEP amplitudes were calculated as the difference between the minimum trough point and maximum peak point within each time window. Time-frequency analysis was performed on the trial-averaged LFP traces using the Morlet wavelet method filtered across various frequencies. Power spectrum analysis was done using a fast Fourier transform function for LFP traces within a time window of 0.5–2 s post visual stimulus, and further quantified by taking the maximum power within various ranges of frequency bands (4–8 Hz, 8–12 Hz, 12–30 Hz, and 30–40 Hz) for each mouse and averaging across mice for comparison between strains.

To obtain single units' raw data were bandpass filtered at 300–6000 Hz, and spike clustering was done using Kilosort. Units were sorted manually to remove any noisy clusters using the Phy interphase. Unit firing rates were used to create peristimulus time histograms with 10 ms bins and smoothed with a 30 ms standard deviation Gaussian kernel. Z-scores were calculated by normalizing the firing rates with the baseline (0–0.5 s) activity ((FR_t_-mean(FR_0-0.5_))/std(FR_0-0.5_)) and averaged across units for line plots. Oscillation duration and frequency were calculated using a peak detection function with a minimum peak height of 0.1 SD from baseline and a minimum separation of 100 ms between peaks. Tuning analysis was done using *Z* score firing rates within the stimulus time window (0.5–0.7 s) to 12 durations:

$$DSI=\frac{\left(Rpref-Rortho\right)}{\left(Rpref+Rortho\right)}$$

Where *OSI = Direction Selectivity Index, R_pref_ = Firing rate to the preferred direction, R_ortho_ = Firing rate to the direction orthogonal to the preferred direction*.

All statistical analyses were done using SciPy and Pingouin Python packages. Oscillation frequency and duration for the unit populations were compared between groups by performing two-sample Kolmogorov-Smirnov tests. The normality of distributions was determined using the Shapiro-Wilk test. For normally distributed data, two-way ANOVA was used, followed by the corresponding Tukey-HSD test, and the Mann-Whitney U test was used for non-normal distributions. Kolmogorov-Smirnov test was used to analyze cumulative distribution functions. All statistical details can be found in the figure legends.

#### Visual discrimination task analysis

Training scores were calculated as the inverse of the cumulative distribution of the percentage of correct touches to the Go stimulus (Hit rate), subtracted by the inverse of the cumulative distribution of the percentage of incorrect touches to No-Go trials (False alarm rate):

TS=zz(Hitrate)−zz(Falsealarmrate)

Where *TS = Training Score, zz = inverse of the cumulative distribution function*.

Once a training score of 2 was reached the mice were considered ‘experts’ in the task. Response times were calculated as the amount of time taken for the mice to touch the screen after stimulus presentation for the Go trials. For non-parametric data Wilcoxon signed-rank test was used for pairwise statistical analysis, and for averaged metrics Mann-whitney U test was used. Kolmogorov-Smirnov test was used to analyze cumulative distribution functions. All statistical details can be found in the figure legends.

#### Three chamber social behavior test analysis

For all recorded videos, DeepLabCut software^30^^,^^31^ was used to track mouse movements, and the corresponding analysis of mouse behavior for the first 60 s of the social test time was done using Python. Mann-Whitney U test was used for non-normal distributions. All statistical details can be found in the figure legends.
