# Supplementary material for: FMRP restoration in parvalbumin interneurons: A circuit-specific improvement of visual learning in fragile X syndrome
Source: iScience. 2025 Nov 21;28(12):114132. doi: 10.1016/j.isci.2025.114132 (PMC12753247; doi:10.1016/j.isci.2025.114132)
Supplement: Document S1. Figures S1–S3. [file mmc1.pdf]

iScience, Volume *28*

Supplemental information

FMRP restoration in parvalbumin interneurons: A circuit-specific improvement of visual learning in fragile X syndrome

Sanghamitra Nareddula, Violeta Saldarriaga, Xinwan Hu, Paige Alyssa Edens, Mia Fehlinger, and Alexander A. Chubykin

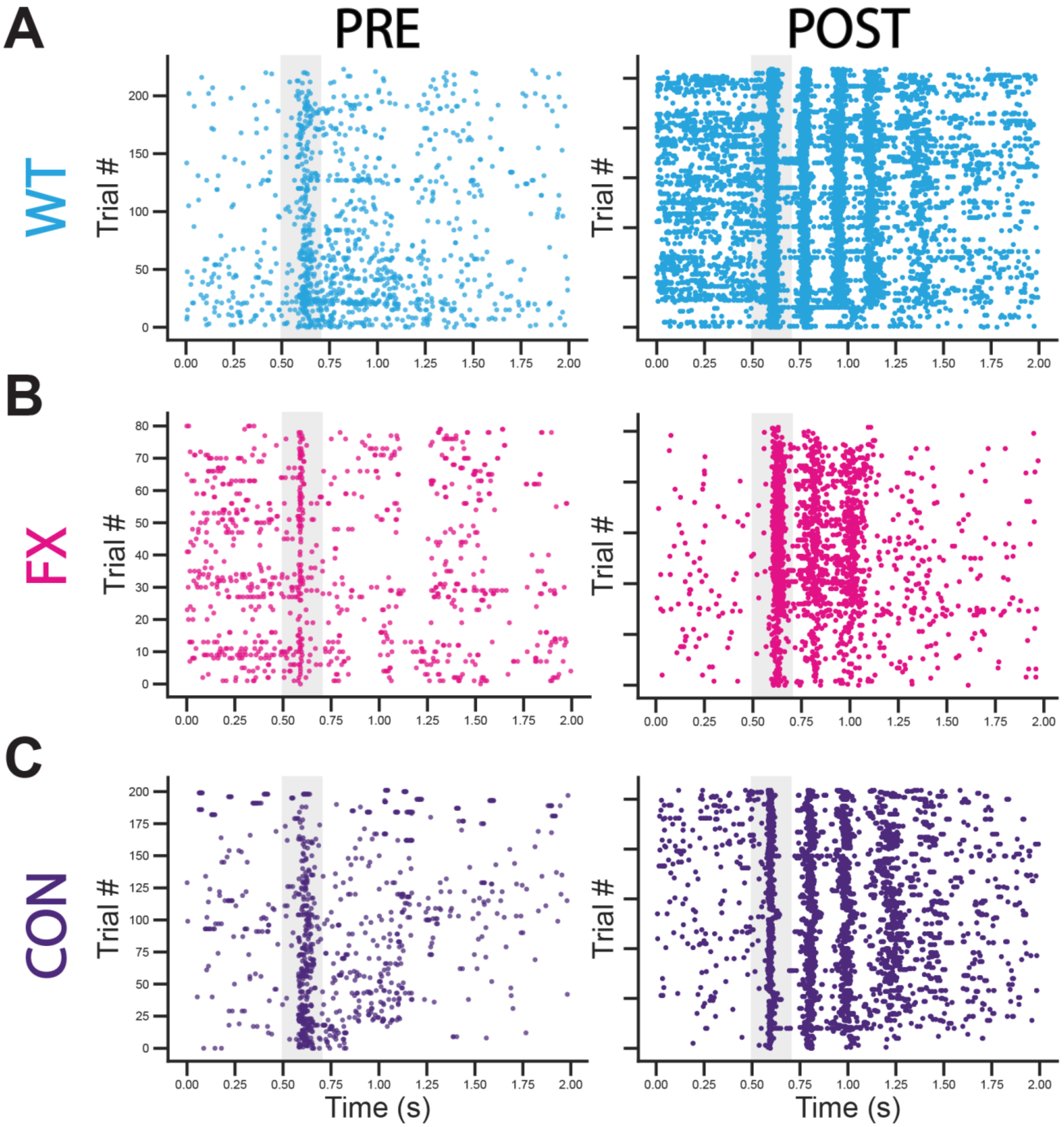

Figure S1. Example unit raster plots showing spike times prior to and after visual training, related to Figure 3.

(A) Example units for pre and post training conditions in WT.
(B) Example units for pre and post training conditions in FX.
(C) Example units for pre and post training conditions in CON.

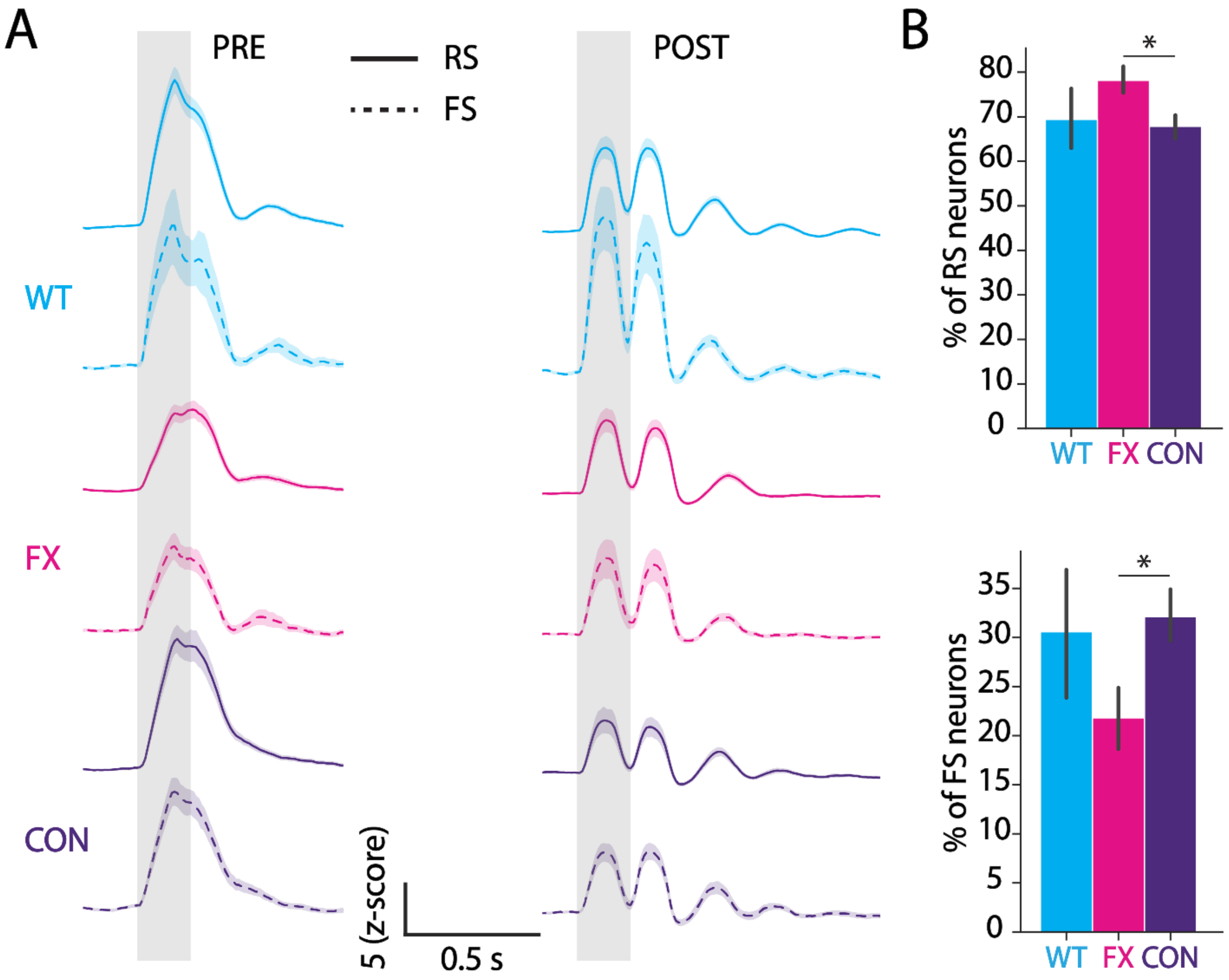

Figure S2. Layer separated unit population activity for fast-spiking (FS) and regular-spiking (RS) neurons, related to Figure 3.

(A) Baseline normalized z-score firing rates averaged across all V1 units shown in (Figure 3A) pre (left) and post (right) visual experience separated by waveform as fast-spiking (FS) and regular-spiking (RS) neurons.

(B) Percentage of active RS (top) and FS neurons (bottom) of the total number of active neurons identified in V1 post visual experience, averaged across mice in each strain. (WT: RS=307 units; FS=104 units, n=13 mice, FX: RS=422 units; FS=131 units, n=12 mice, CON: RS=377 units; FS=181 units, n=15 mice). Mann-whitney U test. (%RS; CON vs FX: $p=0.02$, %FS; CON vs FX: $p=0.02$)
Data are presented as mean ± SEM.
*$p<0.05$.

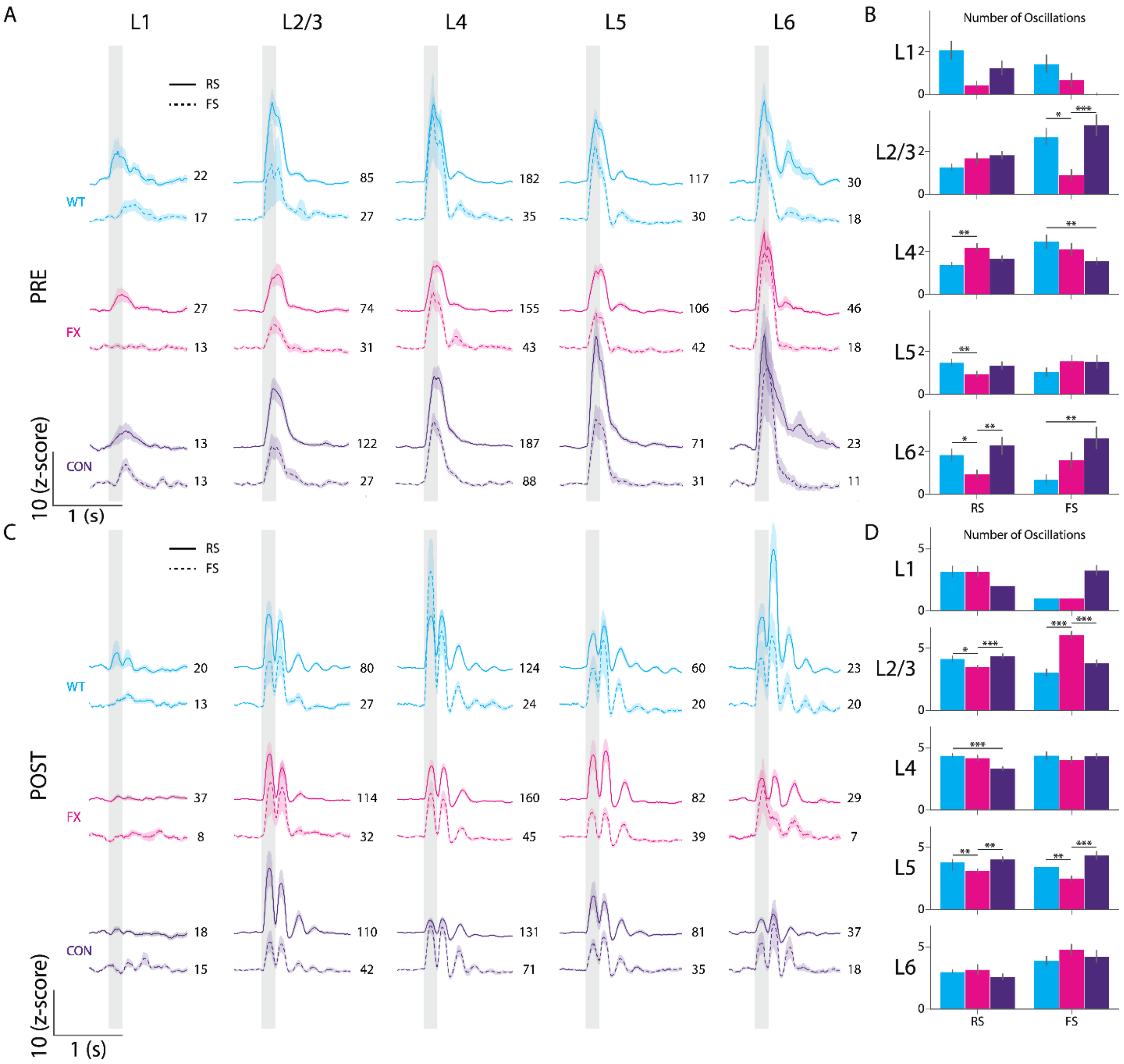

Figure S3. Layer separated unit population activity for fast-spiking (FS) and regular-spiking (RS) neurons, related to Figure 3.

(A) Baseline normalized z-score firing rates averaged across all units shown in (Figure 3A) pre visual experience separated by waveform as FS and RS neurons across all cortical layers in V1 – layer1 (L1), layer2/3 (L2/3), layer4 (L4), Layer5 (L5), and Layer6 (L6). Numbers at end of each trace denote number of units.

(B) Mean number of oscillation cycles for identified peaks from baseline normalized z-score firing rates shown in (A), for FS and RS units across all layers. Mann-whitney U test. (L2/3 FS; WT vs FX: p=0.005, CON vs FX: p=0.00027, L4 RS; WT vs FX: p=0.0014, L4 FS; WT vs CON: p=0.0067, L5 RS; WT vs FX: p=0.006, L6 RS; WT vs FX: p=0.01, CON vs FX: p=0.0098, L6 FS; WT vs CON: p=0.001).

(C) Baseline normalized z-score firing rates averaged across all units shown in (Figure 3A) post visual experience separated by waveform as FS and RS neurons across all cortical layers in V1 – layer1 (L1), layer2/3 (L2/3), layer4 (L4), Layer5 (L5), and Layer6 (L6).

(D) Mean number of oscillation cycles for identified peaks from baseline normalized z-score firing rates shown in (C), for FS and RS units across all layers. Mann-whitney U test. (L2/3 RS; WT vs FX: $p=0.014$, CON vs FX: $p=8.44e-05$, L2/3 FS; WT vs FX: $p=1.82e-06$, CON vs FX: $p=2.72e-05$, L4 RS; WT vs CON: $p=5.76e-06$, L5 RS; WT vs FX: $p=0.0053$, CON vs FX: $p=0.0014$, L5 FS; WT vs FX: $p=0.0078$, CON vs FX: $p=0.0004$)

Data are presented as mean ± SEM.

*$p<0.05$, **$p<0.01$, ***$p<0.001$.
